# Porous PZT Films: How Can We Tune Electrical Properties?

**DOI:** 10.3390/ma16145171

**Published:** 2023-07-22

**Authors:** Liubov Delimova, Dmitry Seregin, Georgy Orlov, Nina Zaitseva, Ekaterina Gushchina, Alexander Sigov, Konstantin Vorotilov

**Affiliations:** 1 Ioffe Institute, 194021 Saint-Petersburg, Russia; ladel@mail.ioffe.ru (L.D.); katgushch@yandex.ru (E.G.); 2 SEC “Technological Center”, MIREA—Russian Technological University (RTU MIREA), 119454 Moscow, Russia; d_seregin@mirea.ru (D.S.); georgiiorlov@mail.ru (G.O.); assigov@yandex.ru (A.S.)

**Keywords:** ferroelectric film, lead zirconate titanate, chemical solution deposition, porosity, polarization, porous boundary, local current

## Abstract

Porous ferroelectric lead zirconate titanate (PZT) films are a promising material for various electronic applications. This study focuses on understanding how the structure-directing agent, polyvinylpyrrolidone, can alter the structure and electrical properties of porous PZT films prepared through chemical solution deposition. Films with various porosities of up to ~40 vol.% and pore connectivities from 3-0 to 3-3 were prepared and studied by capacitance–voltage, dielectric hysteresis, transient current, photocurrent, and local current techniques. We have found that a linear decrease in material volume in a porous film is not the only factor that determines film properties. The creation of new internal grain boundaries plays a key role in changing electrical properties. This research expands the understanding of physical phenomena in porous ferroelectric films and may facilitate the development of new materials and devices.

## 1. Introduction

Manufacturers of ferroelectric ceramics generally aim to increase the density of the ceramic in order to achieve improved electrical performance. Pores and voids formed after high-temperature sintering are considered defects. However, some applications require the introduction of artificial porosity to meet specific material properties. Firstly, porous ferroelectric ceramics are used in hydrophones, where porosity provides a better figure of merit (FOM) and acoustic coupling to water [1]. Another application is IR detectors, where a better FOM results from a decrease in permittivity and volume specific heat [2]. The same reason for choosing porous ceramics (better FOMs due to lower permittivity) has driven a rapidly developing field of energy harvesting based on piezo- and pyroelectric effects and improved dynamic mechanical and electrical performance under shock compression in power supply devices [3,4]. A variety of processing techniques have been proposed to obtain porous ceramics with different internal structures, including polymer sphere burning, the polymer sponge method, freeze casting, and 3D printing, etc. [4,5].

Thin films of ferroelectric ceramics are studied less than their bulk counterparts. The main motivation for introducing porosity in ferroelectric thin films was to enhance film thickness. The film thickness of lead zirconate titanate (PZT) deposited by chemical solution deposition (CSD) is limited to ~30–50 nm due to cracking caused by film shrinkage due to the loss of solvent and organics, as well as crystallization. Repeated cycles of deposition and heat treatment are employed to produce thick films [6]. For example, the coating and baking processes need to be repeated 20 to 30 times to achieve the desired result. A ~2 µm film thickness is required in MEMS devices, which leads to low throughput and high scrap rates in mass production.

Porous films of piezoelectric materials are highly desirable for various electromechanical applications, including transducers, sensors, and energy harvesters [7]. A porous film structure leads to local elastic relaxations and improves electromechanical response [8,9]. In contrast, N. Suzuki observed anisotropic compressive stress in porous BaTiO_3_ films, which leads to an enhanced ferro- and piezoelectric response [10]. A porous structure also has a positive effect on the magnetic characteristics of multiferroic films [9]. In addition, multiferroic behavior may be obtained by impregnating magnetic particles into a porous ferroelectric film [11].

A possible implementation of porous films in memristors could be the creation of artificial synapses with improved stability of filament formation [12,13]. Porous ferroelectric films can also be used to improve photoelectrochemical performance in photoelectrocatalysis, thanks to their enhanced surface area and reactivity [14].

H. Kozuka et al. propose to increase the thickness of ceramic films by incorporating polyvinylpyrrolidone (PVP) in precursor solutions as a stress-relaxing agent [15,16,17]. An idea to use N, N-dialkyl-, or N-alkylcarboxamide precursors in combination with sol-gel hydrolysis and condensation of silicon alkoxide belongs to T. Saegusa and Y. Chujo [18,19]. They have shown that when a silicon-oxide network is formed in the presence of an organic polymer, which is a strong hydrogen acceptor, to form hydrogen bonds, the organic polymer is drawn into the three-dimensional network of silica. This inorganic–organic gel can be transformed into a porous material through the destruction of organic species during heat treatment. H. Kozuka et al. have extended this approach to the preparation of ferroelectric PZT and BaTiO_3_ films. They speculate that in a PVP-modified system, the strong intermolecular interaction between the carbonyl groups (C=O) of PVP and the hydroxyl radicals (OH) of the metal-oxide network can play a critical role in retarding the condensation reaction and promoting stress release [15,17,20].

The introduction of porosity alters the microstructure and electrical properties of ferroelectric films. The main influential factor for applications is the reduction in permittivity across a wide frequency range up to THz [21,22,23]. This reduction enables an increase in pyroelectric performance, for instance [24,25]. Polarization decreases with increasing porosity [26]. However, there are no systematic data on the dependencies of polarization and coercive field on porosity for thin films, as have been obtained for bulk ceramics [27]. Yu. Podgorny et al. reported a larger dead layer thickness at a porous PZT–metal interface and a higher leakage current compared to a dense PZT film [28]. Higher leakages may be caused by various factors, such as chemical defects [29], grain boundary charge carrier transport [30], and carbon residuals [31], and can significantly impact device performance and leakage mechanisms. Therefore, it is important to conduct further studies on this matter.

Thus, despite the fact that porous ferroelectric films have been studied for more than 20 years, there is still no clear understanding of their potential applications due to the absence of systematic studies and models that describe the relationship between electrical properties and porosity. Is this the only way to obtain ceramic films with higher thickness but inferior properties compared to dense films, or is porosity itself useful in meeting specific electrical properties? To provide insight into the electrical properties of porous films, we conducted a comprehensive study on PZT films prepared using the CSD method. The films were prepared with a wide range of PVP content, ranging from 1 to 20 wt.%, in order to obtain films with varying degrees of porosity and internal structure, ranging from isolated pores to highly interconnected ones. The film structure was studied using SEM, XRD, and AFM methods. Characterization of electrical properties includes polarization hysteresis loops and their asymmetry, dielectric constant, polarization dependencies of the transient current and short-circuited photocurrents, and local current distribution. As a result of this study, we have shown that introducing porosity in ferroelectric ceramic films allows for the tuning of their electrical properties and can be utilized in device manufacturing.

## 2. Materials and Methods

A film-forming solution was prepared using zirconium and titanium isopropoxides, and dry lead acetate was obtained directly from PbO, following the method described in [32]. The Zr/Ti ratio was chosen to be 48/52. To compensate for the loss of PbO during the high-temperature treatment, an excess of 14 mol.% Pb was added to the film-forming solution relative to the stoichiometric composition. Polyvinylpyrrolidone (C_6_H_9_ON)_n_, with a molecular weight of 360,000, was used as a porogen. Different amounts ranging from 1 to 20 wt.% of PVP were added to the PZT stock solution, as shown in Table 1. The films were spin-coated onto a 6-inch silicon wafer (100), 10 Ohm*cm, with the following structure: Si-SiO_2_-TiO_2_-Pt, which was produced by Inostek, Korea. The film thicknesses of thermally grown SiO_2_ oxide, sputtered TiO_2_, and Pt layers were 300 nm, 10 nm, and 160 nm, respectively. Spin-on deposition was performed using a spin-coater (WS-650-8NPP; Laurell, MI, USA) at a rotation speed of 2500 rpm for 15 s. The films were grown through repeated cycles of solution deposition and drying. Each deposited layer was dried at 200 °C (soft bake) and 400 °C (hard bake) for 10 min to remove organic residues. After the final layer was deposited, the samples were annealed at a temperature of 650 °C for 15 min to perform crystallization. 

Film thickness (*d*) and refractive index (*n_p_*) were estimated using spectroscopic ellipsometry (Sentech SE-850; Berlin, Germany). The ellipsometry data were fitted using the Bruggemann and Tauc–Lorentz optical models. The optical (volume) porosity, p, of the film was estimated using the Lorentz–Lorentz relationship, which takes into account the refractive indexes of both dense and porous films. Some samples could not be measured by ellipsometry due to excessive light scattering.

Ferroelectric polarization was recorded using an AixACCT TF 2000 (AixACCT, Aachen, Germany). Capacitor voltage dependencies were obtained using an LCR meter (4284A; Agilent, Santa Clara, CA, USA). A mercury probe (model 802-150; MDC Corp, Chatsworth, CA, USA) with an area of 0.53 mm^2^ was used for metal contact connection. Semitransparent Pt electrodes, 10 nm thick, with an area of 10^−3^ cm^2^, were formed by DC magnetron sputtering. These electrodes were used to study the film properties when the applied field was perpendicular to the plane of the film, i.e., between the top and bottom electrodes. These include the asymmetry of hysteresis loops, as well as the polarization dependencies of the transient current and short-circuited photocurrent, which were measured using a Keithley 6487 Picoammeter/Voltage Source (Keithley Instruments, Solon, OH, USA). To excite charge carriers under visible light illumination, we used a Cree XLamp 7090 LED with a wavelength of 457 nm and a pump power of 25 mW/cm^2^ on the film surface. The crystalline structure was studied using a DRON-3 (Burevesnik, Saint-Petersburg, Russia) diffractometer in scanning mode Θ-2Θ, with Cu Kα1 radiation. The microstructure was studied using the scanning electron microscope (SEM) FEI NovaNanoSEM 230 (FEI, Thermo Fisher Scientific, Waltham, MA, USA). Grain size distribution and film porosity were estimated using ImageScope 12.4.6 software. The surface topography and local current distribution in the PZT films were studied using conductive atomic force microscopy (c-AFM) with an Ntegra Aura scanning probe laboratory from NT-MDT Co., Zelenograd, Russia.

## 3. Results

### 3.1. General Characterization of the Film Thickness and Microstructure

As film thickness affects its electrical properties, two groups of samples were prepared: the first group consisted of films with a thickness of about 400 nm (referred to as “thin” films), while the second group consisted of films with a thickness in the range of 600–700 nm (referred to as “thick” films). Detailed information, including PVP content, the number of applied layers, refractive index, volume porosity calculated from the refractive index, film thickness, and thickness per one layer deposition, is collected in Table 1.

**Figure 1:**
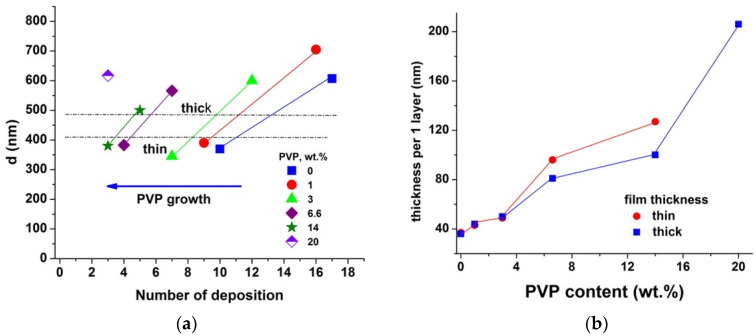
Film thickness vs. number of applied layers (**a**) and thickness of one applied layer against PVP content (**b**).

Figure 1 shows the relationship between film thickness and the number of applied layers at different PVP content (a), as well as the thickness of one applied layer against PVP content (b). Film thickness increases with PVP content. As a result, a thicker film can be obtained with fewer deposition steps, as illustrated in Figure 1a.

**Figure 2:**
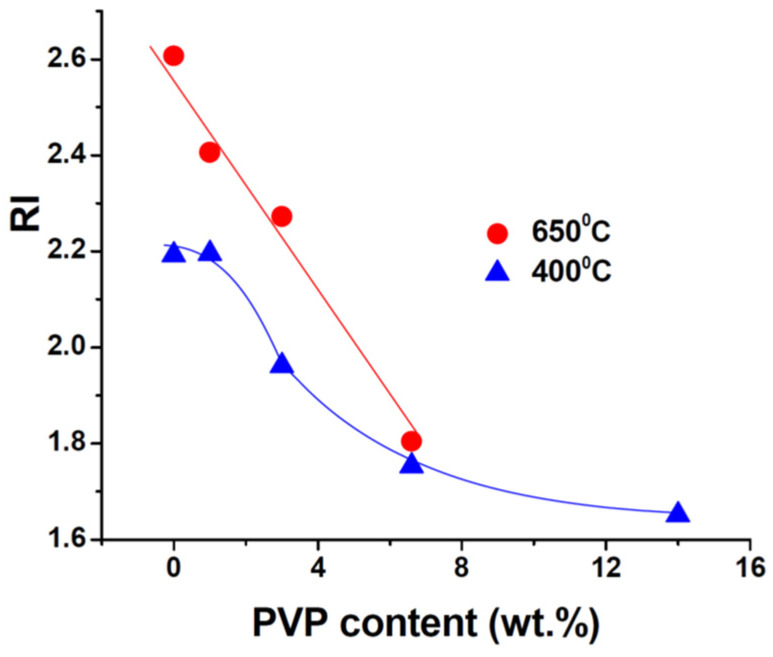
Refractive index vs. PVP content, measured before (400 °C) and after (650 °C) crystallization.

**Figure 3:**
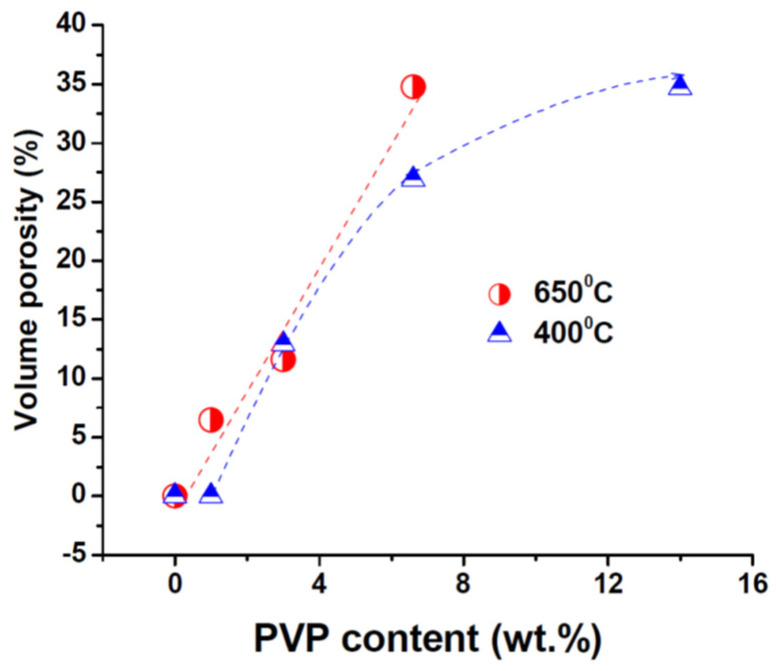
Volume porosity vs. PVP content.

Figure 2 shows the dependencies of the refractive index on PVP content. Two types of measurements were carried out on the samples: data collected before crystallization of the structure correspond to annealing at 400 °C, and data obtained after crystallization refer to annealing at 650 °C. The refractive index decreases with PVP content due to an increase in porosity. Volume porosity calculated using the Lorentz–Lorentz relationship is shown in Figure 3. Volume porosity increases almost linearly up to 6.6 wt.% PVP content. Higher PVP content complicates ellipsometry measurements due to light scattering. However, data obtained after annealing at 400 °C before crystallization started show a further increase in porosity for a film with 14 wt.% PVP content.

**Figure 4:**
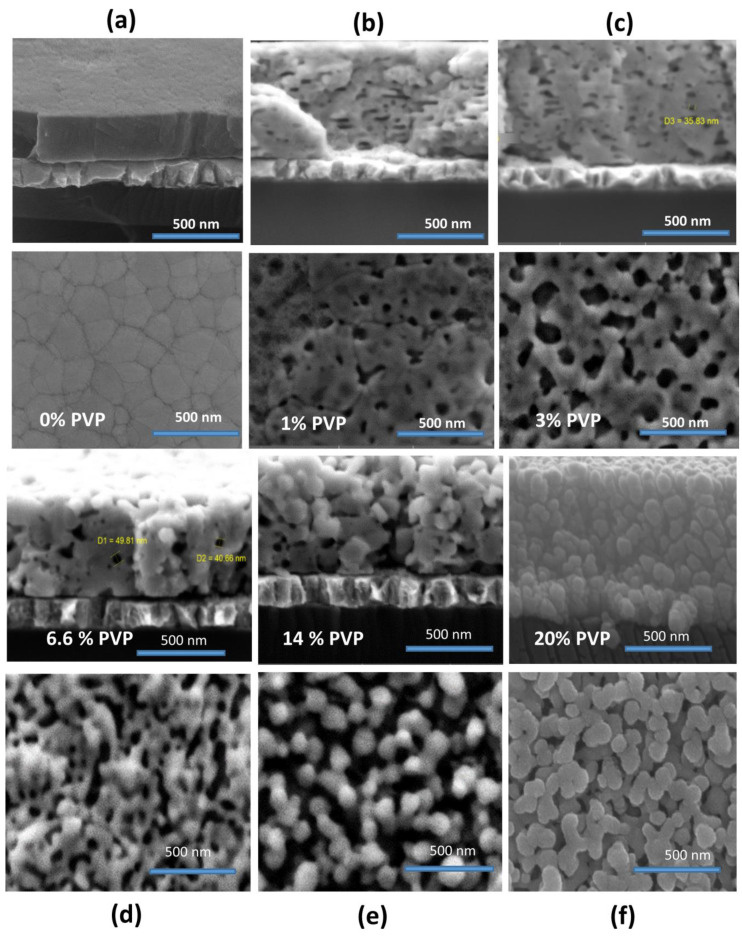
Cross-sectional (**top**) and in-plane (**bottom**) SEM images of PZT films prepared with different PVP content, wt.%: 0—(**a**); 1—(**b**); 3—(**c**); 6.6—(**d**); 14—(**e**); 20—(**f**).

Figure 4 shows cross-sectional (top) and in-plane (bottom) SEM images of a dense PZT film (a) and PZT films with varying PVP content (b)–(f). The initial dense PZT film exhibits a pronounced columnar grain structure, which is typical for PZT grown on (111) Pt [29]. The addition of 1 to 3 wt.% PVP induces isolated pores within the perovskite grains, as seen in Figure 4b,c, which can be referred to as 3-0 connectivity [33]. Detailed descriptions of the film’s microstructure can be found in Ref. [34]. A higher PVP content results in a significant transformation of the film structure. With an excess of 6.6 wt.% PVP content, films lose their columnar grain structure and become polycrystalline. They consist of fairly fine grains, approximately 50 nm in size. They are characterized by irregular and highly interconnected pores, as can be seen in Figure 4d–f. This percolated structure can be attributed to 3-3 connectivity [33].

**Figure 5:**
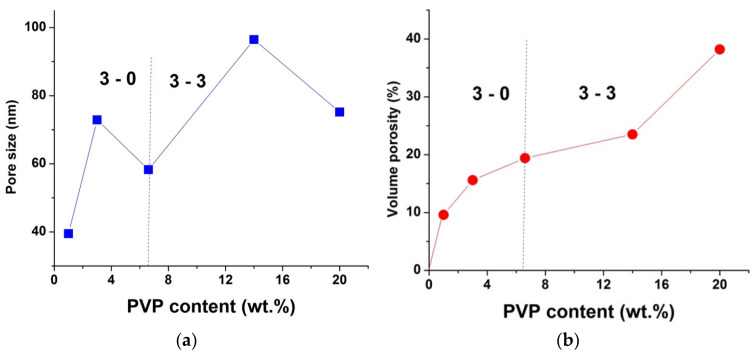
In-plane SEM results: pore size on the left (**a**) and porosity on the right (**b**), depending on PVP content. Data obtained by statistical processing of in-plane SEM pictures in ImageScope software.

Statistical analysis of plane-view SEM images reveals that there is an increase in both the average pore size and volume porosity as PVP content increases (refer to Figure 5). However, at 6.6 wt.%, there is a decrease in the mean pore size, which may be attributed to reaching the percolation threshold and the emergence of narrow interconnecting mouths (also known as ink-bottle pores) and internal voids [35].

### 3.2. X-ray Diffraction Study

**Figure 6:**
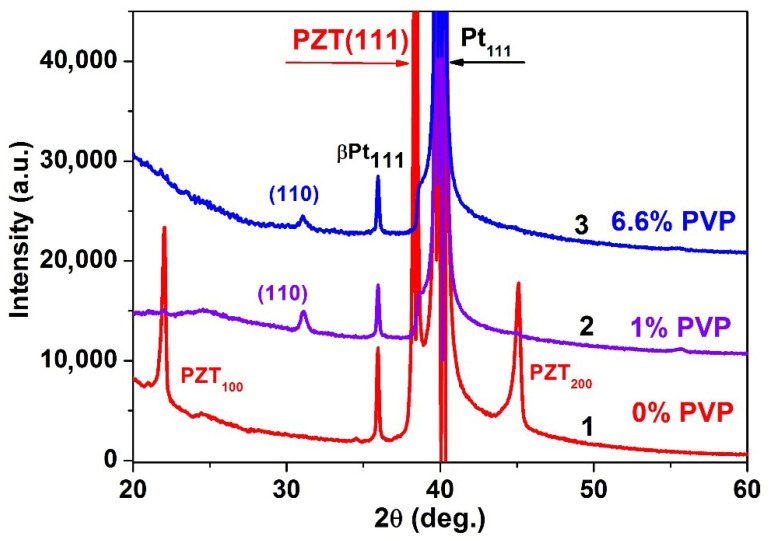
X-ray diffraction patterns of PZT films prepared with different wt.% PVP: (1)—0; (2)—1; (3)—6.6.

X-ray diffraction (XRD) data confirm the transformation of the crystal structure. Figure 6 presents XRD patterns measured in a dense PZT film (curve 1) and those prepared with PVP content of 1 wt.% (curve 2) and 6.6 wt.% (curve 3). It is observed that the dense film exhibits a predominant (111) Pe texture along the volume diagonal and a weaker (100) direction, which is characteristic of columnar grain films grown on (111) Pt. In porous PZT films, the strong columnar grain texture is destroyed and changes to a predominant Pe (110) and weaker (211) orientations. The peak intensity decreases with increasing porosity.

Ratios of the reflection intensities obtained from the diffraction peaks are listed in Table 2.

Lattice parameters, calculated from XRD-measured interplane distances assuming a pseudo-cubic lattice, are presented in Table 3 (JCPDS International Centre for Diffraction Data). In a dense film, the lattice constants found are smaller than the value for an unstressed PZT powder, indicating the existence of compressive stresses in the dense PZT film. However, in porous films with increasing porosity, the pseudo-cubic lattice parameter increases. For the (110) reflection, the stress is already removed at 3 wt.% PVP, while for the (211) reflection, the stress decreases but still remains at 14 wt.% PVP. In general, the measured lattice constantly increases with PVP content, which provides evidence for stress relaxation. This is the reason for the improved resistance of porous films to cracking.

### 3.3. Dielectric Constant, Polarization, and Coercive Field

**Figure 7:**
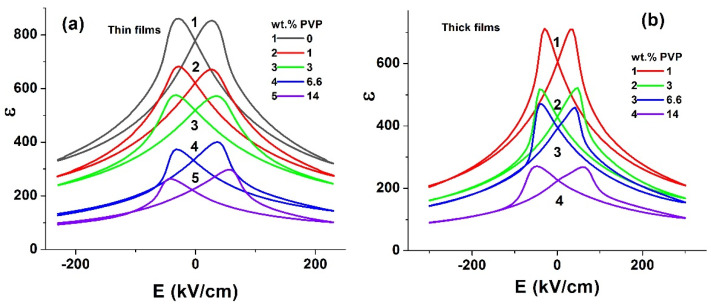
Dielectric constant vs. electric field in thin (**a**) and thick (**b**) PZT films.

Figure 7 presents dielectric constant vs. electric field, found from capacitance–voltage (CV) curves measured from porous PZT films. These dependencies are typical for PZT films and demonstrate a decrease in permittivity with increasing PVP content, while maintaining the characteristic shape.

**Figure 8:**
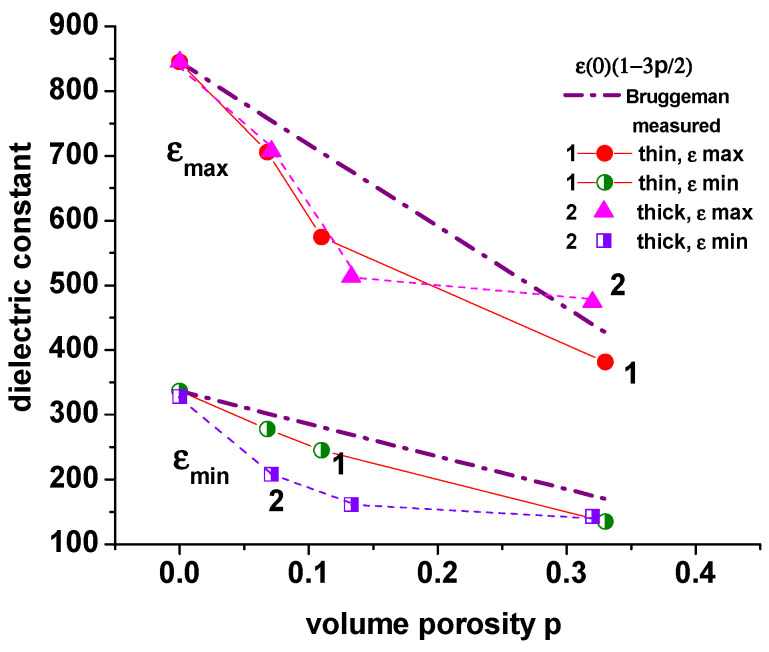
Dielectric constants ε_max_ and ε_min_ as a function of volume porosity measured as curves: (1) thin films and (2) thick films; dashed lines correspond to the calculated Brüggemann’s approximation for pores with 3-3 connectivity.

Maximum and minimum values of permittivity, obtained from CV curves depending on volume porosity (estimated from optical measurements), are plotted in Figure 8. Brüggemann’s approximation for binary composites is commonly used to estimate effective permittivity, considering volume porosity *ε*(*p*) = *ε*(0)·(1 − 3*p*/2), where *ε*(*p*) is the permittivity of a porous film, *ε*(0) represents the permittivity of a dense film, and p represents the porosity [36]. The approximation is seen to give larger values compared to measured ones (Figure 8), indicating the existence of additional factors contributing to the reduction in dielectric constant, such as loss of texture, changes in perovskite grain size, and distribution of local fields.

The operation of most electronic devices that use ferroelectric film is based on the presence of spontaneous polarization and its ability to be switched by an electric field. Therefore, retaining the magnitude of polarization and the symmetry of the hysteresis loop is a crucial factor in determining the performance and reliability of a device. To investigate the asymmetry of hysteresis loops, we employed the Sawyer–Tower method, along with a depolarization procedure. In this procedure, a decaying sinusoidal voltage was applied to the bottom electrode of a Pt/PZT/Pt structure, which was connected in series with a 220 nF reference capacitor. Polarization was measured by the charge on a reference capacitor [37]. This method enables the film to be transferred to its initial depolarized state, establishing a reference point on the charge axis. This reference point allows for the detection of any potential asymmetry in polarization switching when an applied bias is applied.

**Figure 9:**
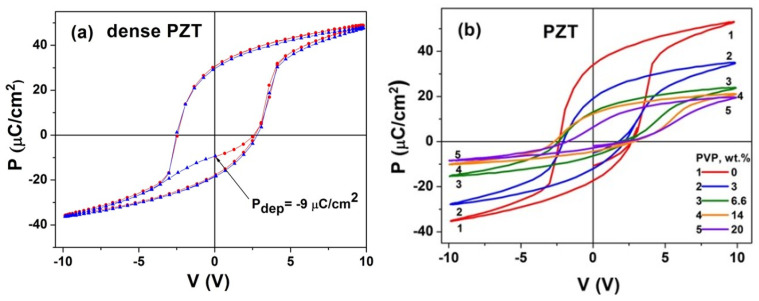
Polarization vs. sample voltage in dense (**a**) and porous PZT films (**b**).

Hysteresis loops, measured in a depolarized state by applying three half periods of sinusoidal voltage with an amplitude of ±10 V and frequency of 64 Hz, are shown in Figure 9a for a dense PZT film. It is observed that the measured hysteresis loops are strongly asymmetric. The significant excess of positive remanent polarization compared to the negative case indicates that the PZT film has an unswitchable positive polarization, which manifests itself in a depolarized state as the value of *P_dep_* = −9 µC/cm^2^. This is due to the presence of stresses in the film [37].

Hysteresis loops measured in a depolarized state in porous PZT films are shown in Figure 9b. It is also observed that the growth of porosity results in a decrease in the magnitude of polarization and hysteresis area, as well as tilting of the loops and a reduction in their rectangularity, starting from 20 wt.% PVP. Similar results were reported for PZT ceramics in [27]. Increasing porosity also leads to a loss of saturation in the bird beak region due to an increase in leakage [38,39,40].

**Figure 10:**
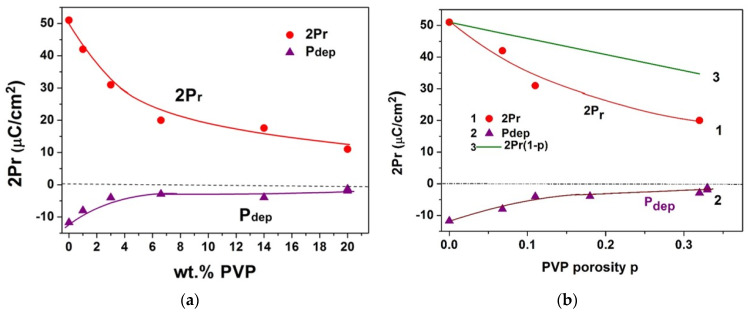
Switchable polarizations 2*P*r and unswitchable polarization *P*_dep_ vs. weight percent of PVP content (**a**) and volume porosity (**b**).

Figure 10 shows switchable polarization (2*P*r) and polarization in the depolarized state (*P*_dep_) as functions of the weight percent of PVP content (a) and volume porosity (b).

W. Wersing et al. [36], with Marukate’s effective medium approximation [41], have found an approximation that adequately describes the polarization in porous ceramics: *P* = *P*_0_ (1 − *p*), where *P*_0_ is the polarization of dense ceramics. Y. Zhang et al. [27], studying polarization-electric field behavior of ferroelectric materials, introduce a depolarization factor (*d_P_*): *P = d*_P_·*P*_0_ (1 − *p*). They obtained *d_P_* in the range of 0.5–0.8 depending on pore configuration and alignment.

It can be seen that switchable polarization in PZT films decreases with porosity at a faster rate than the linear reduction predicted by Wersing’s approximation, as shown by curve 3 in Figure 10b. The depolarization factor was estimated to be within the range of 0.9–0.59. This finding is consistent with the results obtained for ceramics [27,42] and suggests the existence of an additional depolarization factor that is linked not only to a reduction in the volume of ferroelectric material. At the same time, the value of *P*_dep_ associated with nonswitchable polarization also decreases with increasing PVP, indicating stress relaxation in the porous film.

**Figure 11:**
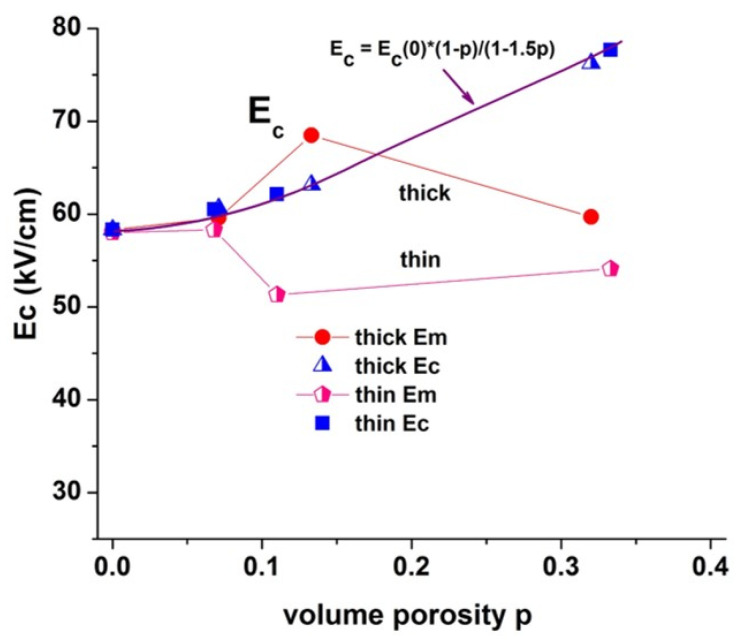
Dependences of coercive field vs. volume porosity in the measured PZT films.

The value of coercive field *E*_C_ extracted from the hysteresis loops in both thin and thick films as a function of volume porosity *p* is shown in Figure 11. According to W. Wersing et al. [36], in a bulk porous PZT ceramic coercive field, *E*_C_ has to increase monotonically as *E*_C_ = *E*_C_(0)·(1 − *p*)/(1 − 3*p*/2), where *E_C_*(0) is the coercive field of dense ceramics, as shown in Figure 11. Interestingly, the measured films do not show a sufficient increase in coercive field.

### 3.4. Polarization Dependences of the Transient Current

To investigate the relationship between porosity and the behavior of the polarization vector, we examine polarization dependencies of the transient current in porous PZT films. Previously, we experimentally demonstrated that pronounced current peaks can appear in the current–voltage (*I*–*V*) curves of epitaxial or well-textured polycrystalline PZT films with nonconductive grain boundaries when the polarization vector and external bias directions coincide [30]. To elucidate the nature of these peaks, we developed a model that describes the non-stationary transport of charge carriers in an n-type PZT film. In this model, electrons, which are produced by oxygen vacancies, are trapped by Ti^+3^ levels [43,44] and subsequently move between these levels in response to an electric field [45]. The model and the performed numerical simulation showed that a necessary condition for a current peak to appear is the presence of an electron-accumulated space charge layer (SCL) near that electrode, where a significant positive uncompensated polarization charge has formed. Therefore, observation of the current peak indicates that the polarization vector maintains its magnitude and direction consistently across the entire film thickness, extending up to the electrodes. Note that a pronounced current peak can appear in the *I*–*V* curves of a well-textured PZT film with conductive grain boundaries when an external bias is applied against the polarization vector [30].

During experiments, bias was applied in a sequence of steps, each with an amplitude of 0.1 V and a duration of 0.2 s. The current was recorded at the end of each step. The voltage changed from 0 to 3 V and then returned to 0. In the measurement circuit, the domain-switching time is ~2 ms. Therefore, by the time the current was recorded, the current caused by domain switching had already passed through the external circuit. The recorded current is transient, meaning that it is associated with the movement of mobile charge carriers and variations in the electric field over time.

**Figure 12:**
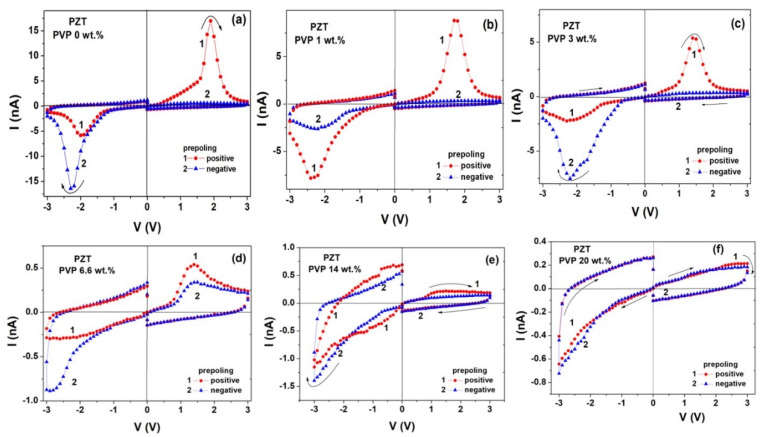
Current–voltage curves measured at different pre-polarizations in PZT films with varying PVP content, wt.%: (**a**)—0; (**b**)—1; (**c**)—3; (**d**)—6.6; (**e**)—14; (**f**)—20.

For both bias directions, measurements were carried out with different initial polarizations. Before each measurement, the film was depolarized and then polarized in a specific direction by applying a bias pulse of V = ±6 V for 2 s. Figure 12 displays the current–voltage curves measured in PZT films with varying PVP content under positive (curve 1) and negative (curve 2) preliminary polarizations. For the dense PZT film, curves 1 and 2 exhibit pronounced current peaks (±16 nA) when the directions of bias and the polarization vector coincide (Figure 12a). Similar current peaks, but with lower current values, are observed for PZT films with 1–3 wt.% PVP content and pore connectivity of 3-0 (Figure 12b,c). However, with an excess of 6 wt.% PVP, both pronounced peaks and their dependence on the polarization direction actually disappeared (Figure 12d–f). More details are provided in Table 4.

Indeed, above 6 wt.% PVP content, there are no more peaks at a negative bias, possibly because the value of the negative remanent polarization −*P*r is too small to induce an accumulated SCL at the bottom electrode. At a positive bias, an intermediate situation takes place: there are no peaks in 42% of all structures, and a weak current peak appears either at *P* > 0 in 35% or at *P* < 0 in 23% of the structures, the latter corresponding to a conductive grain boundary [30]. At 14 wt.% PVP at *V* > 0, there are no peaks in 70% of all structures; only a weak plateau appears either at *P* > 0 in 15% or at *P* < 0 in 15% of the structures. At 20 wt.% PVP at *V* > 0, half of the structures have no peak; the other half show a weak plateau at *P* > 0.

### 3.5. Polarization Dependences of the Short-Circuited Photocurrent

Measuring a photocurrent under short-circuit conditions is a well-known method for studying internal electric fields in a film. The time-dependent photocurrent of the short-circuited capacitor M/PZT/M was measured under different pre-polarization conditions. First, the film was depolarized and then polarized in the dark using a bias pulse of ±6 V for 2 s. After the bias was switched off, the external circuit was shorted. When the transient dark current decreased to zero, the film was illuminated with visible light with a wavelength of 457 nm (2.7 eV). The photocurrent flowing in the external circuit was then recorded at 1 s intervals.

**Figure 13:**
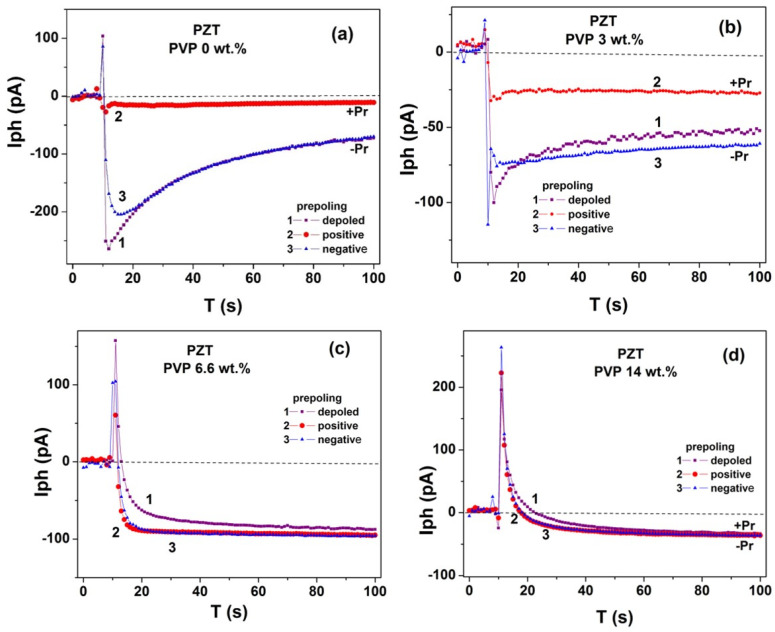
Dependencies of the photocurrent on illumination time at different pre-polarizations in PZT film with PVP content, wt.%: (**a**)—0; (**b**)—3; (**c**)—6.6; (**d**)—14.

Figure 13 presents the photocurrents measured in the dense (a) and porous (b–d) PZT films at various pre-polarizations. Initially, there is a burst of photocurrent, which then relaxes to a stationary value. In the depolarized state of the dense film, a negative stationary photocurrent flows downward from the top electrode to the bottom one, indicating the presence of a downward built-in averaged electric field in the film (curve 1 in Figure 13a). Positive polarization significantly reduces the magnitude of the negative stationary photocurrent, seen in curve 2, by 59 pA. On the other hand, negative polarization has almost no effect on the magnitude of the photocurrent, seen in curve 3. Nevertheless, the significant disparity in the photocurrent values measured in the positive and negative directions of polarization indicates a noticeable impact of the polarization vector on the photocurrent in a dense film.

It is observed in Figure 13b that at 3 wt.% PVP, the photovoltaic current exhibits all the distinctive characteristics of the dense film photocurrent: a negative initial burst, relaxation to a stationary negative value, and the same polarization effect. However, the influence of polarization on the current magnitude is weaker. Indeed, positive pre-polarization reduced the stationary photocurrent of the depoled state by only 25 pA (curve 2). Additionally, there was a difference of 34 pA between the photocurrents measured at positive and negative pre-polarizations (curve 3 in Figure 13b). When the PVP content exceeds 6 wt.%, there is an initial positive burst of photocurrent, followed by relaxation to a negative stationary value. This negative value is no longer dependent on polarization, as shown in Figure 13c,d. Moreover, as seen in Figure 13d, the value of the stationary negative photocurrent decreases with increasing porosity. This indicates a weakening of the average built-in electric field in the film.

### 3.6. Local Current Study by c-AFM

Local current distributions were studied using the c-AFM method, which enables simultaneous measurement of a film’s surface topography and local current map. Rigid (stiffness coefficient ~5–20 N/m) conductive probes with a wear-resistant diamond-coated cantilever were used for imaging, which retained their conductive properties even when a voltage of ±10 V was applied. The interaction force between the c-AFM probe and the surface under study is approximately F ≈ 300–500 nN. The probe tip radius is ~100 nm, and the radius of the contact area is ~15 nm. An external bias of ~6–7 V was applied to the bottom electrode, while the top electrode was grounded.

**Figure 14:**
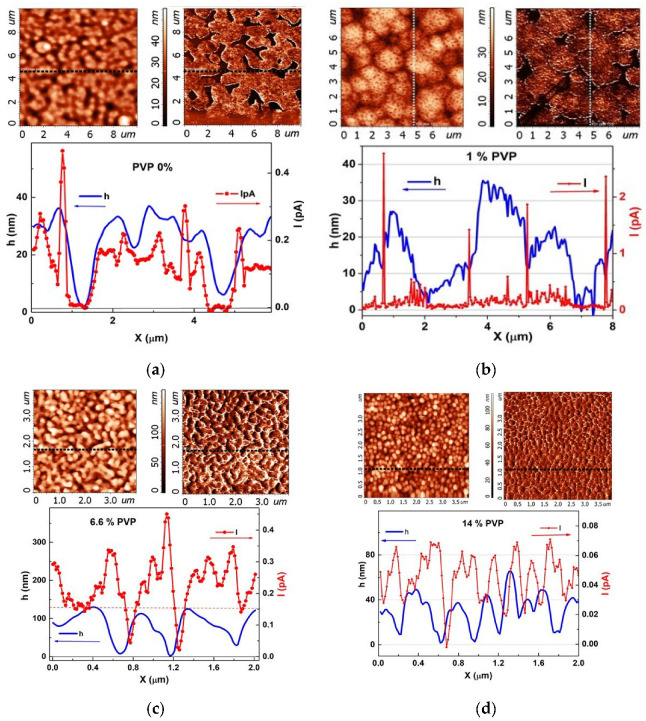
The results of the c-AFM study for PZT films with varying PVP content, wt.%: (**a**)—0; (**b**)—1; (**c**)—6.6; (**d**)—14. For each PZT film, 2D images (**upper left**) of the topography and (**upper right**) maps of local currents obtained by scanning the PZT film are shown; below are the profiles of topographic signal h (**left** scale, solid line) and local current I (**right** scale, circles) flowing along the profile shown above by the dotted line.

Figure 14 shows the results of the c-AFM study on PZT films with different PVP content. For each PZT film, 2D images of the topography and local current maps obtained by scanning the PZT film are shown, along with the topographic signal and local current flowing along the selected profile in the images. Figure 14a shows that in the dense film, the white areas of increased conductivity in the local current map are correlated with the light areas in the 2D image of the topography, which correspond to grain heights. The grain width falls within the range of 0.15–0.4 μm; the minima in the topographic signal correspond to the grain boundaries. It can be observed that variation in the local current closely mirrors the topography, with distinct current peaks occurring within the individual grains. In addition, zero-current positions correlate with the grain boundaries, thereby confirming that the grain boundaries are non-conductive. One can say that in the dense film, local currents flow inside the grains, forming a dense columnar structure.

In films containing 1 wt.% PVP, individual peaks of the intergrain current are observed, which significantly exceed the very low current flowing within the grains (see Figure 14b). When exceeding 6 wt.% PVP, the distribution of the local current changes drastically (Figure 14c,d). It can be observed that variations in the local current no longer follow the profile of the grain relief. Instead, the current sharply increases in the regions of grain boundaries and pore boundaries.

## 4. Discussion

### 4.1. Structure—Polarization

The film thickness with a single spin-on deposition increases by almost fivefold with the addition of 20 wt.% PVP; see Figure 1b. The main reason is the increase in the viscosity of the film-forming solution. According to Meyerhofer’s approximation for spin-on deposition, film thickness *d*~*ν*^1/3^, where *ν* is the viscosity of the solution [46]. No cracking was observed in any samples studied in this research. The addition of a stress-relaxing agent, polyvinylpyrrolidone (PVP), to the PZT precursor solution leads to an increase in film thickness and prevents film cracking [15,16,20]. Kozuka et al. observed that the tensile stress in BST films is greatly reduced with increasing PVP content.

According to a study of the silicate sol-gel process with PVP by Saegusa and Chujo, PVP forms hydrogen bonds with oxoalkoxides, slowing down the polycondensation reaction [18,19]. Kozuka et al. suggest an interaction between PVP and PZT precursors via carbonyl groups (C=O) and hydroxyl bonds (OH) [15]. However, the synthesis of a film-forming solution in our case does not assume any water addition for hydrolysis (see Section 2). Lead acetate is also free of water according to its direct preparation from PbO [32]. We assume that intermolecular interaction between PVP and PZT oxoalkoxide occurs via the donor–acceptor mechanism.

Film thickness is increased without any saturation region up to 20 wt.% PVP content (Figure 1b). In contrast, the addition of the surfactant Brij, used as a porogen for preparation of silica and PZT porous films, demonstrates a saturation region for film thickness–porogen content dependencies, as there are no sufficient terminal hydroxyls to provide interaction between the Brij and the metal-oxide network [34]. This fact limits the porosity of films prepared by EISA with Brij surfactant [34]. In contrast, the PVP-based process can produce higher porosity for PZT films. The porosity value obtained from ellipsometry data increases with PVP content in the range 1–6.6 wt.%, reaching about 32% (Figure 3). A further growth of PVP content leads to an increase in porosity. Indeed, the refractive index of PZT film before crystallization shows a further decrease (Figure 2), while the calculated value of porosity increases (Figure 3). A rough estimation from in-plane SEM image analysis supports a porosity increase in the range of PVP content from 1 to 20 wt.% up to the value of about 40%; see Figure 5b.

The films demonstrate a rather high average pore size of 40–100 nm (Figure 5a). This value coincides with the data obtained by transmission electron microscopy in [34]. It should be noted that the EISA process with Brij precursor provides a smaller pore size and a uniform pore distribution [34].

The SEM study showed a columnar grain structure of the dense PZT film, isolated disk-shaped pore inclusions inside the PZT grains at 1 to 3 wt.% PVP (3-0 connectivity), and a percolated porous structure when exceeding 6.6 wt.% PVP. In the latter case, the porous structure becomes continuous and is attributed to 3-3 connectivity. The X-ray diffraction data confirm the crystal structure transformation: the strong columnar grain Pe (111) texture in the dense film is destroyed and changes to a predominant Pe (110) and weaker (211) in porous films. The lattice constants in the dense film are less than in PZT powder, indicating the presence of compressive stress. However, in porous films, the lattice constant increases with PVP content, which provides evidence for stress relaxation. This explains the better cracking resistance of porous films.

The *CV* curves of all samples demonstrate nonlinear butterfly-like behavior typical of ferroelectrics; see Figure 7. All films also demonstrate polarization hysteresis (Figure 9). Introducing porosity decreases electrical nonlinearity but the ferroelectric behavior persists even in the case of high film porosity. Both the maximum permittivity value representing the contribution of domain wall movement near the coercive field, and the minimum permittivity value related to low contribution of domain walls due to their electric field clamping, show a decrease with increasing porosity (Figure 8). However, Brüggemann’s approximation for binary composites gives larger values of effective permittivity compared to the experimental data (Figure 8). Dielectric hysteresis loops also show a faster decrease in remanent polarization than predicted by Wersing’s approximation [36]; see Figure 10. This indicates that, in addition to the decrease in volume of ferroelectric material, which is considered in these models, there are other factors that affect electrical properties. Among them are texture destruction, an emergence of additional interfaces and defects that cause depolarization fields, and domain wall clamping.

Domain wall movement in ferroelectric materials is influenced by various factors, such as crystal structure, defects, grain boundaries, and the distribution of the local electric field. According to finite-element analysis in Ref. [47], local electric field distribution plays a main role in porous ferroelectric ceramics. It showed that increasing the pore angle relative to the direction of the applied electric field decreases the local electric field. This results in a reduced domain wall dynamic and a broadening of the distribution of switching times. This is the main reason for the observed dependence of polarization on porosity.

On the other hand, the relaxation of mechanical stresses in porous films leads to a decrease in nonswitchable polarization *P*_dep_ [37]; see Figure 10. We can suggest that this is a reason for the weak dependence of the coercive field on porosity (Figure 11) in contrast to the monotonic growth with porosity predicted in [36].

**Figure 15:**
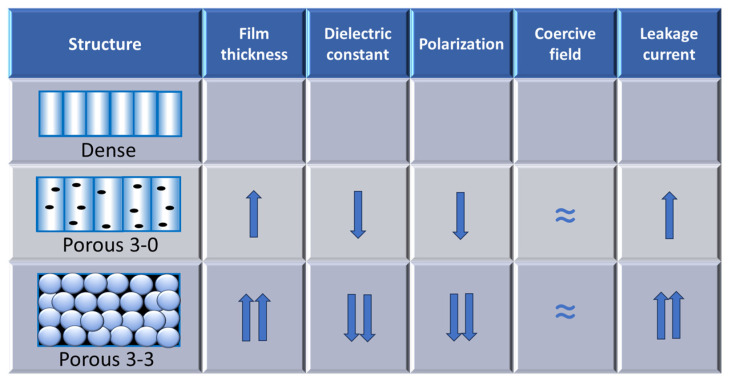
A simplified scheme illustrates the main trends in the relationship between the structure of porous PZT films and their main electrical properties.

Summarizing the discussion in these sections, Figure 15 illustrates the primary trends in the correlation between the structure of porous films and their key electrical properties. In general, an increase in porosity alters the film structure from columnar grains with distinct 3-0 inclusions to sponge-like uniaxial grains with 3-3 interconnected pores. As a result, the film thickness increases, the dielectric constant decreases, polarization decreases, the coercive field does not change significantly, and the leakage current increases.

### 4.2. Polarization Dependences of the Transient Current

A depolarized Pt/PZT/Pt structure contains two Schottky barriers with depleted SCLs created by the positive charge of oxygen vacancies and a quasi-neutral region between them, where the positive charge of oxygen vacancies is compensated by the negative charge of electrons captured by Ti^+3^ atoms. As shown in [41], at the contact of a polarized film where a negative polarization charge arises, the thickness of the depleted SCL increases, while at the opposite contact it decreases, and if the polarization exceeds a certain level, then the depleted SCL at this contact is replaced by an accumulated layer. When an external bias is applied in the direction of the polarization, the electric field begins to pull electrons from the accumulated SCL and transfer them to the depleted SCL at the opposite contact, compensating for the charge of vacancies and thereby reducing the resistance of this layer and the entire film. This causes an increase in the current and the formation of a current peak. When the accumulated layer has exhausted all electrons, further electrons taken from the already depleted layer increase the thickness of this SCL and, hence, the film resistance, which leads to a decrease in the current and completes the formation of the current peak. Thus, there are several factors necessary to produce a current peak: (1) the presence of an accumulated SCL and (2) coincidence of the directions of the applied bias and the polarization vector.

In the dense PZT film, all necessary conditions are present; therefore, we can observe the highest current peaks in both bias directions. At 1–3 wt.% PVP, there are pronounced current peaks, but with a lower current value. The decrease in electron density of the accumulated layer can be explained by the reduction in polarization as porosity increases. A current peak is formed by electrons from the accumulated layer hopping over Ti levels within the grains. When pores are introduced into the PZT matrix, a new current component appears that is associated with carrier hopping through deep levels along grain boundaries and pore boundaries. It is unlikely that this current contributes to the formation of current peaks because the dependence of the current on the polarization direction is not visible.

At a PVP content of 6.6 wt.%, the film structure experiences significant distortion. This leads to the appearance of numerous depolarizing fields associated with charged pore boundaries and significant variations in both direction and magnitude of the electric field throughout the film. Consequently, there is a general decrease in the magnitude of polarization, the emergence of poled areas with different poling directions, and even the presence of unpoled areas [48]. As a result, the average built-in electric field responsible for the transfer of carriers between the contacts is weakened, while conductive grain boundaries emerge. The disappearance of current peaks can be explained by the simultaneous influence of a decrease in polarization, a weakening of electron transfer across titanium levels, and competitive conductivity along grain boundaries.

Table 5 illustrates the main mechanisms of charge transfer in porous PZT films, as obtained from transient current measurements.

### 4.3. Polarization Dependencies of Photocurrents

We assume that when illuminated by visible light with a photon energy less than the band gap PZT, an electron in the valence band absorbs an energy quantum and is excited to the Ti level located at 1 eV below the bottom of the conduction band, while a free hole appears in the valence band. The electrons excited from the Ti level to the conduction band fall back to the Ti level or move in the electric field to the contact in a much shorter time than 1 s; therefore, they do not contribute to the measured photocurrent. The initial burst of photocurrent is defined by the direction of the built-in electric field in the film. In a dense PZT film, the built-in field is most likely to be a downwards electric field of the bottom Schottky barrier. Therefore, the initial burst of photocurrent is negative. Further relaxation of the photocurrent to its stationary value reflects the establishment of an equilibrium between the processes of photoexcitation, recombination, and charge carrier transport. As a result, in the depolarized state, the stationary negative photocurrent density is 7 × 10^−7^ A/cm^2^ at an illumination intensity of 0.025 W/cm^2^. This magnitude agrees well with a photovoltaic current value of 4.7 × 10^−7^ A/cm^2^ for a 210 nm thick PLZT film at an illumination intensity of 0.05 W/cm^2^, which is reported in [49].

The effect of preliminary polarization on the photocurrent can be explained by the bulk photovoltaic effect, in which a photocurrent is induced in the polarization direction. In the case of +*P*r = 24 µC/cm^2^, it seems that the action of the bulk photovoltaic effect almost completely compensates for the action of the built-in electric field associated with Schottky barriers and polarization charges. As a result, the negative photocurrent decreases to −11 pA. In the case of −*P*r = −9 µC/cm^2^, a contribution to the negative photocurrent can occur due to the bulk photovoltaic effect, while a positive photocurrent can appear due to a depolarizing field or field enhancement of the top Schottky barrier. As results for −*P*r, the induced photocurrent is too small to change significantly the negative stationary photocurrent of the depolarized state.

A film with 3 wt.% PVP retains all the characteristics of the dense film photocurrent: a negative burst followed by relaxation to a stationary negative value, and the same dependence on polarization. However, the influence on the current magnitude is weaker due to the decreased polarization value caused by porosity. However, when the PVP concentration exceeds 6 wt.%, the initial burst of photocurrent changes direction from negative to positive. The film is illuminated from the side of the top electrode, where a higher density of excited carriers can occur due to non-uniform light absorption across the film thickness, as well as light scattering associated with the presence of pores. Therefore, during the initial illumination moment, the majority of the excited carriers are subjected to the influence of the positive electric field of the top Schottky barrier, resulting in a surge of positive current. This is then followed by a relaxation period, leading to a steady-state negative photocurrent. Moreover, the magnitude of the stationary photocurrent decreases with increasing porosity. This indicates a weakening of the average built-in electric field in the film, which is practically independent of the polarization direction.

Analyzing the photovoltaic current measurements, one can draw the following conclusions. Charged pore boundaries in films with 3-3 connectivity lead to the appearance of numerous depolarizing fields. This not only decreases the magnitude of polarization but also results in the loss of a single direction for the polarization vector throughout the film.

### 4.4. Local Current Study

**Scheme 1:**
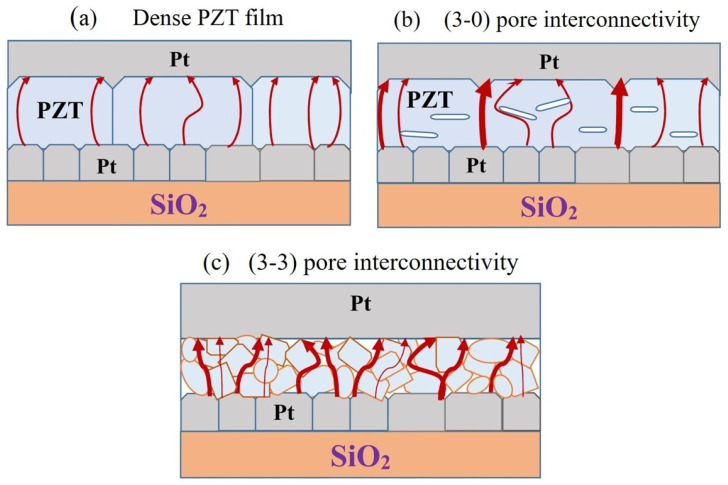
Sketch illustrating the current flowing across the dense (**a**) and porous PZT films with pore interconnectivity (3-0)—(**b**) and (3-3)—(**c**). The red arrows indicate the currents flowing through the grains (thin) and along the boundaries of grains and pores (thick). White color indicates air pores.

A dense PZT film with a columnar grain structure contains grains and grain boundaries. The c-AFM study showed that in the dense PZT film, a local current flows between the bottom and top electrodes through the PZT grains, while the grain boundaries are non-conductive. Scheme 1a is a sketch illustrating the current flowing through the dense PZT film.

The introduction of pores into a PZT film creates a new type of interface—pore boundaries. Inside the film, there are grains, grain boundaries, and pores within the grains. A pore may appear between grains, but it is still considered part of the grain boundary. Furthermore, since a polarization charge can exist at the grain boundaries, the pore boundaries can also be charged. It cannot be ignored that carbon residues are the cause of enhanced charge transport at the grain boundaries, despite the high annealing temperature (650 °C) for PZT film crystallization. In the case of porous organosilica films used in the back-end-of-line process, the presence of carbon residuals on the pore walls is a cause of increased leakage currents [50]. However, it is difficult to detect and clean these residuals, and this issue requires further study.

The resulting pore boundaries create new pathways for the flow of electric current. Electrons move either inside the PZT grain or along pore boundaries or grain boundaries. At low PVP content below 6.6 wt.% (3-0 connectivity), individual intergrain current peaks are observed, which significantly exceed the very low current flowing within the grains (see Scheme 1b). At a higher PVP content (3-3 connectivity), the grain size decreases. However, the number of current pathways along the pore boundaries increases. As a result, the currents flowing along the pore boundaries exceed those flowing inside the grains, as shown in Figure 14c,d and Scheme 1c. As a result, conductivity and leakage currents in the porous films increase compared to the dense film.

## 5. Conclusions

Porous ferroelectric lead zirconate titanate (PZT) films are a promising material for various applications, including MEMS, transducers, sensors, energy harvesters, IR detectors, memristors, photoelectrocatalysis, etc. This research focuses on understanding the structural and electrical properties of porous ferroelectric PZT films. Chemical solution deposition with the addition of 1 to 20 wt.% of polyvinylpyrrolidone as a structure-directing agent was used to obtain films with different levels of porosity and internal structure. These films ranged from isolated pores to highly interconnected spongy–granular structures. We have found that existing models for binary composites, which only consider the linear decrease in material volume in a porous film, do not fully account for all the factors that govern film properties. The creation of new internal grain boundaries plays a key role in changing electrical properties. This results in the emergence of depolarizing fields and fluctuations in both the orientation and intensity of the electric field across the film. The latter, in turn, leads to a general decrease in the magnitude of polarization, and the direction of the polarization vector is not maintained consistently across the thickness of the film. The pore boundaries are new pathways for the flow of electric current. The number of these pathways increases with increasing porosity, resulting in currents flowing along the pore boundaries that exceed those flowing inside the grains. As a result, leakage current in porous films increases compared to a dense film. We suggest that understanding the structural peculiarities and physical processes in porous PZT films opens up new opportunities for their applications in electronics. Future work includes the engineering of materials with a predetermined pore structure, interfaces, and inclusions in order to obtain new material properties for various applications.

## Figures and Tables

**Table 1:** General characteristics of porous thin and thick (bold font) PZT films.

PVP wt.%	Volume Porosity, %	Number of Layers	Thickness *d*, nm	Refractive Index	Thickness per Layer
0	0	10	370	2.54	37
**0**	**0**	**17**	**607**	**2.61**	**36**
1	6.8	9	390	2.40	43
**1**	**7.1**	**16**	**705**	**2.39**	**44**
3	11.0	7	345	2.29	49
**3**	**13.3**	**12**	**600**	**2.23**	**50**
6.6	33.3	4	383	1.83	96
**6.6**	**32.0**	**7**	**566**	**1.85**	**81**
14	*	3	380	*	127
**14**	*	**5**	**500**	*	**100**
**20**	*	**3**	**617**	*	**206**

* Ellipsometric measurements were not meaningful because of light scattering.

**Table 2:** Percentage ratios of the reflection intensities found from diffraction peaks in porous PZT films.

PVP wt.%	XRD Texture
0	(111) 100% (100) 28% (200) 22%
1	(110) 100% (211) 14%
3	(110) 100% (211) 7%
6.6	(110) 100% (211) 16%
14	(110) 100% (211) 5%
20	(110) 100%

**Table 3:** Lattice parameters in porous PZT films.

**XRD reflexes**	**100**	**110**	**111**	**200**	**211**
**PZT powder reflex, %**	20%	100%	20%	22%	26%
**Lattice constant, Å**	4.068	4.068	4.068	4.068	4.068
**PVP, wt.%**	**Lattice constant in PZT, Å**				
**0**	4.029		4.061	4.019	
**1**		4.061			4.047
**3**		4.072			4.027
**6.6**		4.072			4.055
**14**		4.072			4.062
**20**		4.073			

**Table 4:** Characteristics of the *I*–*V* curves in polarized PZT films with different PVP content.

PVP, wt.%	*I*–*V* PEAK Is Observed When:		
0	*P*//*V*, nonconductive grain boundaries (GBs)		columnar grain structure
1	*P*//*V*, 100% nonconductive GBs		columnar grain structure; porous 3-0 connectivity
3			
6.6	*V* < 0	there are no peaks, 100%	porous 3-3 connectivity causes a destruction of the columnar grain structure; numerous depolarizing fields induced by polarization charges at pore boundaries lead to a decrease in polarization and disappearance of current peaks
	*V* > 0	there are no peaks, 42%	
		35% weak peaks at *P* > 0, nonconductive GBs	
		23% weak peaks at *P* < 0, conductive GBs	
14	*V* < 0	there are no peaks, 100%	
	*V* > 0	there are no peaks, 70%	
		15% weak plateau at *P* > 0	
		15% weak plateau at *P* < 0	
20	*V* < 0	there are no peaks, 100%	
	*V* > 0	there are no peaks, 50%	
		50% weak plateau at *P* > 0	

**Table 5:** Charge carrier transfer mechanisms in porous PZT films.

	Polarization, *P*	Current Way	Grain–Boundary Conductivity	*I*–*V* Peak	Reason
Dense film	*P* conserves its direction and value across the film up to electrodes	Inside the grains by the action of an averaged built-in field	Nonconductive	Pronounced *I*–*V* peaks in both bias directions	Columnar grain texture; *P* is high enough to induce an accumulated space charge layer at a cathode
Porous 3-0	*P* value decreases, but conserves its direction across film	Inside the grains; a small intergrain current appears	Nonconductive	Pronounced *I*–*V* peaks with a lower current value	Columnar grain texture; porous 3-0 connectivity
Porous 3-3	*P* value decreases; *P* vector loses its single direction across a film	Grain boundaries and along pores	Conductive	*I*–*V* peaks disappear due to *P* decreasing; growing conduction along GBs and pores	Sponge-like 3-3 pore connectivity

## Data Availability

Data are contained within the article. The raw data presented in this study are available on request from the authors.
